# Identification of Bis(methylsulfanyl)methane and Furan-2(5*H*)-one as Volatile Marker Compounds for the Differentiation of the White Truffle Species *Tuber magnatum* and *Tuber borchii*

**DOI:** 10.1021/acs.jafc.4c00714

**Published:** 2024-04-17

**Authors:** Philipp Schlumpberger, Martin Steinhaus

**Affiliations:** † TUM School of Natural Sciences, Department of Chemistry, Technical University of Munich, Lichtenbergstraße 4, 85748 Garching, Germany; ‡ Leibniz Institute for Food Systems Biology at the Technical University of Munich (Leibniz-LSB@TUM), Lise-Meitner-Straße 34, 85354 Freising, Germany

**Keywords:** white truffle, *Tuber magnatum*, *Tuber borchii*, bis(methylsulfanyl)methane, furan-2(5*H*)-one, untargeted volatilomics, automated solvent-assisted flavor evaporation (aSAFE), comprehensive two-dimensional gas chromatography (GC×GC), stable isotopically substituted compound

## Abstract

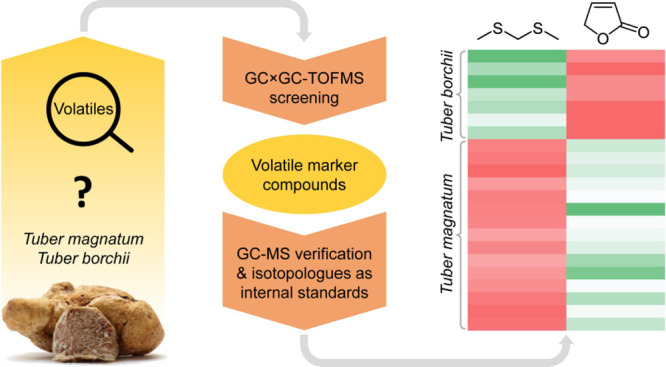

Some truffles are expensive and, therefore, are prone to food fraud. A particular problem is the differentiation of high-priced *Tuber magnatum* truffles from cheaper *Tuber borchii* truffles, both of which are white truffles with similar morphological characteristics. Using an untargeted approach, the volatiles isolated from samples of both species were screened for potential marker compounds by comprehensive two-dimensional gas chromatography–time-of-flight mass spectrometry (GC×GC–TOFMS) and statistical analysis of the obtained semiquantitative data. Results suggested bis(methylsulfanyl)methane and furan-2(5*H*)-one as compounds characterizing *T. magnatum* and *T. borchii*, respectively. Exact quantitation of both volatiles by conventional one-dimensional gas chromatography–mass spectrometry in combination with stable isotopologues of the target compounds as internal standards confirmed both as marker compounds. The method is suitable to be used in the routine analysis for the objective species differentiation of *T. magnatum* and *T. borchii*.

## Introduction

Truffles are the hypogeous fruiting bodies of some ascomycete fungi, in particular those of the genus *Tuber* in the Tuberaceae family.^1,2^ Their hyphae form an ectomycorrhiza with the roots of various trees and shrubs such as oaks, elms, poplars, hazels, willows, pines, and cedars.^2−5^ The symbiotic relationship serves to exchange nutrients between the fungus and the plant.^3^

The fruiting bodies of all of the *Tuber* species are edible. According to their color, these are typically classified as white or black truffles. Both are highly appreciated, particularly in French, Spanish, Italian, and Greek cuisines. Whereas black truffles are used in the kitchen mainly as funds or essences, white truffles are typically not cooked but added in thin slices to the finished dish. Risotto, pasta, pizza, omelet, salad, and polenta are just some popular dishes that are commonly served with a topping of white truffle slices.^3,6^

Only two species predominantly share the white truffle market: *Tuber magnatum* Picco, yielding the Alba truffle or Piedmont truffle, and *Tuber borchii* Vittad., previously named *Tuber albidum*, yielding the Bianchetto truffle.^1,3,5,7^ Both species are native to Europe. The occurrence of *T. magnatum* is not limited to the Piedmont region of Italy, but this species is also found in other Italian regions, in Croatia, Hungary, Romania, and Slovenia.^1,2,5^ Italy is also the center of occurrence of *T. borchii*; however, its growing region extends over an even larger geographical area than that of *T. magnatum* and includes England, Finland, Germany, Hungary, Poland, and Switzerland.^5,8^

Truffles are among the most expensive foods. This particularly applies to *T. magnatum*.^5^ An increased demand in combination with a declining crop has caused the prices to rise substantially in recent years.^3,4^ In January 2024, the price of a *T. magnatum* truffle ranged from 2000 to 3000 $. *T. borchii* truffles were significantly cheaper and cost ∼250 to 700 $.^9^ The higher price of *T. magnatum* truffles is associated with their stronger and richer aroma–a major factor for their great popularity–and with difficulties in their cultivation.^1,6^ Whereas other species, including *T. borchii*, are successfully cultivated in truffle plantations, cultivation of *T. magnatum* has rarely been effective to date.^3,4,10^

In contrast to the substantial differences in price and availability, the appearances of *T. magnatum* and *T. borchii* truffles are often very similar. *T. magnatum* truffles are typically 2–6 cm in size but can reach up to 15 cm.^11^ The surface is smooth and usually pale ochre in color.^11,12^ Reddish spots occasionally occur.^12^ A cross-section of a *T. magnatum* truffle shows light brown flesh with white veins.^5^*T. borchii* truffles, on average, are smaller than *T. magnatum* truffles and typically show sizes of 2–3 cm.^11^ The surface is just as smooth and pale ochre-colored as the surface of *T. magnatum* truffles; reddish spots are also often present.^5,11^ The flesh may be darker in color and the veins may be wider compared to *T. magnatum* truffles.^5,11^

Given the morphological similarities between *T. magnatum* and *T. borchii* truffles in combination with a high morphological variability within each species, it is often impossible to unequivocally assign an individual truffle to one or the other species on the basis of the morphology alone. Consequently, the risk of cheap and readily available *T. borchii* truffles being marketed as *T. magnatum* truffles is high. The vast price difference makes selling *T. borchii* truffles fraudulently labeled as *T. magnatum* truffles a lucrative business. Chemical marker compounds characterizing each species would be beneficial to counteracting this type of food fraud.

Studies on the differentiation of truffle species based on chemical analyses have already been published.^13−19^ A widely applied approach is based on untargeted metabolomics using liquid chromatography (LC) in combination with mass spectrometry (MS).^20,21^ For example, Li et al.^20^ were able to differentiate the European truffle species *T. melanosporum* and the four Chinese truffle species *T. indicum*, *T. panzhihuanense*, *T. sinoaestivum*, and *T. pseudoexcavatum* by a comprehensive LC–MS profiling including the analysis of amino acids, saccharides and nucleosides, alkaloids, flavonoids, carnitines, organic acids, phenols, alcohols, esters, and sulfur compounds. Individual marker compounds, however, were not determined. Creydt and Fischer^21^ concentrated on the analysis of the lipidome. In the two white truffle species included in the study, namely, *T. magnatum* and *T. borchii*, they identified 33 metabolites. The analysis of 26 of these metabolites proved to be suitable for distinguishing between the two white truffle species.

Another approach that has been evaluated for the differentiation of truffle species is the analysis of volatiles by gas chromatography (GC) in combination with MS. Pelusio et al.^22^ compared the white truffle species *T. magnatum* and the black truffle species *T. melanosporum* on the basis of volatile sulfur compounds analyzed by headspace solid phase microextraction (HS–SPME) and GC–MS. (Methylsulfanyl)methane, (methyldisulfanyl)methane, dimethyltrisulfane, and bis(methylsulfanyl)methane were identified as important organosulfur volatiles; however, these were not suitable to differentiate between the two species when samples of different harvest years were considered. Kiss et al.^23^ used a modified Likens-Nickerson apparatus to isolate the volatiles from Hungarian black truffles of the species *T. aestivum* and *T. brumale*. GC–MS analysis identified 102 and 104 volatiles in *T. aestivum* and *T. brumale*, respectively. Semiquantitative data revealed apparent differences in the composition of the volatile fraction between the two species. Whereas in *T. aestivum*, 2-methylbutan-1-ol was the predominating volatile followed by hexadecanoic acid, 2-phenylethan-1-ol, butan-2-ol, 1-octen-3-ol, and 2-methylpropan-1-ol, the most abundant volatiles in *T. brumale* were methoxybenzenes such as 1,4-dimethoxybenzene, 1-methoxy-3-methylbenzene, and foremost 1,2,4-trimethoxybenzene. D’Auria et al.^24^ compared the volatiles in three samples of the common white truffle species *T. borchii* with the volatiles in four samples of the rare white truffle species *T. asa-foetida* by HS–SPME–GC–MS. Among the 12 volatiles identified, six compounds including 2-methylpropan-1-ol, 3-methylbutanal, and 3-methylbutan-1-ol were common to both species, whereas 3-methylthiophene, xylene, α-pinene, 3,7-dimethylocta-1,3,6-triene, 3-acetyl-1-propyl-5,6-dihydro-2-naphthol, and 9-(diphenylmethylidene)-9*H*-fluorene were only identified in *T. borchii* and butan-2-one, tetrahydrofuran, benzene, butan-2-yl formate, 2-methylbutan-1-ol, and toluene were only found in *T. asa-foetida*. Among them, however, only 3-methylthiophene and toluene were suitable as marker compounds for *T. borchii* and *T. asa-foetida*, respectively, as only these two compounds were identified in all samples of the particular species. Zhang et al.^25^ compared a sample of a black Chinese truffle with a sample of a white Chinese truffle. Volatiles were isolated by solvent extraction and solvent-assisted flavor evaporation (SAFE), and analyzed by comprehensive two-dimensional gas chromatography in combination with time-of-flight MS (GC×GC–TOFMS). Fifty-eight and 47 volatiles were identified in the black and white truffles, respectively. The authors suggested that the approach might be suitable to discriminate between the two species.

Only two studies were available that compared the volatiles in the two white truffle species, *T. magnatum* and *T. borchii*. Mauriello et al.^26^ analyzed the volatilome of 11 different truffle species, including five samples each of *T. magnatum* and *T. borchii* by HS–SPME–GC–MS. The *T. magnatum* samples were characterized by the presence of bis(methylsulfanyl)methane, (methylsulfanyl)methane, (methyldisulfanyl)methane, and (methyltrisulfanyl)methane, whereas numerous volatiles were identified in the *T. borchii* samples that were absent in the *T. magnatum* samples, including 1,3-xylene, 2-benzothiophene, 2-methylbutan-1-ol, 2-methylbuta-1,3-diene, 2-methylfuran, 2-methylpropan-1-ol, 3-methylbutan-1-ol, 3-methylthiophene, (3*Z*)-3,7-dimethylocta-1,3,6-triene, butan-2-ol, butan-2-yl formate, decane, ethanol, ethenylbenzene, octan-3-one, penta-1,2-diene, pentanal, tetradecanal, tetradecane, and toluene. Gioacchini et al.^27^ analyzed the volatiles of six different truffle species, including *T. magnatum* and *T. borchii*, by HS–SPME–GC–MS. The mass spectral data of the individual runs were processed into an average mass spectrum. Specific mass-to-charge ratio (*m*/*z*) values were assigned to different compound classes, such as alcohols, aldehydes, etc., and their intensities were successfully used for species differentiation.

In summary of the literature overview, differentiation of *T. magnatum* and *T. borchii* truffles on the basis of the analysis of volatile marker compounds seemed feasible. However, previous studies suffered from some deficiencies. For example, structure assignments were only based on mass spectral libraries and were not confirmed by analysis of authentic reference compounds. Semiquantitative data obtained in the untargeted approaches were not confirmed by exact quantitations, e.g., using GC–MS in combination with isotopically substituted internal standards. Furthermore, in the HS–SPME–GC–MS approaches, it remained unclear whether the suggested marker volatiles were genuine truffle constituents or thermal artifacts formed in the hot injector during desorption from the fiber. Accordingly, our study aimed to use the recently developed automated solvent-assisted flavor evaporation (aSAFE) approach^28^ to reproducibly prepare artifact-free volatile isolates from numerous *T. magnatum* and *T. borchii* truffle samples, screen the volatiles for potential marker compounds by GC×GC–TOFMS analysis in combination with statistical analysis of the semiquantitative data, unequivocally assign the structures of the potential marker compounds, and finally verify the marker compounds by exact quantitation using GC–MS analysis in combination with isotopologues of the target compounds as internal standards.

## Materials and Methods

### Truffle Samples

Truffle samples with confirmed authenticity (17 × *T. magnatum*, samples M 01 C – M 17 C; 6 × *T. borchii*, samples B 01 C – B 06 C) were provided by a specialized retailer (La Bilancia, Munich, Germany). Their authenticity was assessed based on unequivocal morphological characteristics. Truffles with an equivocal morphology and truffles with damages were excluded. Truffle samples with unconfirmed authenticity (2 × *T. magnatum*, samples M 18 U and M 19 U*;* 1 × *T. borchii*, sample B 07 U) were obtained from Internet shops. All truffle samples were collected between 2018 and 2020. The fresh material was shock-frosted with liquid nitrogen and stored at–24 °C before analysis.

### Reference Compounds and Stable Isotopically Substituted Volatiles

Isotopically unmodified reference compounds bis(methylsulfanyl)methane and furan-2(5*H*)-one were purchased from Merck (Darmstadt, Germany). (^2^H_8_)Naphthalene was also from Merck. (^2^H_8_)Bis(methylsulfanyl)methane was synthesized as detailed in the literature.^29^ (^2^H_2_)Furan-2(5*H*)-one was synthesized according to a procedure published previously^30^ but with some modifications. In brief, 3a,4,7,7a-tetrahydro-4,7-epoxy-2-benzofuran-1,3-dione (abcr, Karlsruhe, Germany) was deuterated with sodium borodeuteride (Cambridge Isotope Laboratories, Tewksbury, MA, USA) under an argon atmosphere. Acidic workup led to the intermediate (3,3-^2^H_2_)-3a,4,7,7a-tetrahydro-4,7-epoxy-2-benzofuran-1(3*H*)-one, which was extracted by dichloromethane instead of chloroform and then purified by chromatography (3 cm column diameter) on silica gel 60 (0.040–0.063 mm; VWR, Darmstadt, Germany; 60 g). After being washed with *n*-hexane/ethyl acetate (50/50, v/v; 150 mL), the compound was eluted with *n*-hexane/ethyl acetate (25/75, v/v; 150 mL) and ethyl acetate (200 mL). The solvents were removed in vacuo, and the purified (3,3-^2^H_2_)-3a,4,7,7a-tetrahydro-4,7-epoxy-2-benzofuran-1(3*H*)-one was heated to 150 °C at 15 mbar to distill off the target product (5,5-^2^H_2_)furan-2(5*H*)-one (96% purity by GC–flame ionization detector) obtained in a retro-Diels–Alder reaction.

### Organic Solvents

Dichloromethane was obtained from CLN (Freising, Germany) and freshly distilled through a column (120 cm × 5 cm) packed with Raschig rings. Ethyl acetate was obtained from J. T. Baker (Phillipsburg, NJ, USA) and *n*-hexane from Merck.

### GC×GC–TOFMS Analysis

Truffles were cooled with liquid nitrogen and ground in the frozen state with a laboratory mill GrindoMix GM200 (Retsch, Haan, Germany) at 10,000 rpm (2 × 3 s). Dichloromethane (50 mL) and (^2^H_8_)naphthalene (1 μg) were added to the powder (2 g). Under ice-cooling, anhydrous sodium sulfate (6 g) was added, and the mixture was homogenized with an Ultra-Turrax T25 (IKA, Staufen, Germany) at 13,500 rpm for 20 s. After continuous stirring overnight at room temperature and under light exclusion, the mixture was filtered through a folded filter, and the residue was washed with dichloromethane (10 mL). The combined extracts were subjected to aSAFE at 40 °C using a valve open/closed time combination of 0.2 s/10 s.^28^ The obtained volatile fraction was concentrated to a final volume of 1 mL using a Vigreux column (50 × 1 cm) and a Bemelmans microdistillation device.^31^ For each truffle sample, a duplicate or triplicate workup was performed.

The truffle volatile isolates were stored in amber glass vials at −24 °C prior to analysis with a GC×GC–TOFMS system. This was equipped with a 6890 gas chromatograph (Agilent, Waldbronn, Germany), a Combi PAL autosampler (CTC Analytics, Zwingen, Switzerland), a Cooled Injection System (CIS) 4 (Gerstel, Mülheim/Ruhr, Germany), and a DB-FFAP GC column, 30 m × 0.25 mm i.d., 0.25 μm film thickness (Agilent) in the first dimension (^1^D). The end of this column was connected to a dual-stage quad-jet thermal modulator (Leco, Mönchengladbach, Germany) which cryofocused the volatiles in the eluate with the help of liquid nitrogen and transferred them in portions to the column in the second dimension (^2^D), which was a DB-1701 column, 3 m × 0.18 mm i.d., 0.18 μm film (Agilent) and installed in the secondary oven located inside the primary oven. The end of the second column was connected to the inlet (250 °C) of a Pegasus III TOFMS (Leco). Helium at a constant flow of 2.0 mL/min served as the carrier gas. The injection volume was 1 μL. The injection mode was splitless. The initial temperature of the primary oven was 40 °C (2 min), followed by a gradient of 6 °C/min to 95 °C (5 min), a gradient of 3 °C/min to 155 °C, and a final gradient of 4 °C/min to 230 °C (5 min). The initial temperature of the secondary oven was 70 °C (2 min), followed by a gradient of 6 °C/min to 125 °C (5 min), a gradient of 3 °C/min to 185 °C, and a final gradient of 6 °C/min to 250 °C (5 min), resulting in a total run time of 58 min. The modulator was operated with a temperature offset of +50 °C relative to the secondary oven temperature. The modulation period was 4 s, with a hot pulse time of 1 s. Mass spectra were generated in electron ionization (EI) mode at 70 eV with a scan rate of 100 spectra/s and a scan range of *m*/*z* 35–300. The temperature of the transfer line was 250 °C and the temperature of the ion source was 230 °C. Data files were recorded by ChromaTOF software (Leco). Each truffle volatile isolate was analyzed in triplicate.

Data preprocessing and statistical analysis was accomplished by using ChromSpace and ChromCompare+ software (Markes International, Llantrisant, United Kingdom). At first, GC×GC–TOFMS raw data were imported into ChromSpace. Preprocessing started with retention time alignment to compensate for retention time shifts. One chromatogram with medial retention times (*t*_R_) was manually selected as a reference, and the algorithm adjusted the *t*_R_ of all other chromatograms in both the first dimension (^1^*t*_R_) and the second dimension (^2^*t*_R_). The aligned chromatograms were subjected to integration by means of a tile-sum algorithm. In brief, the entire chromatogram was divided into overlapping tiles in size of 120 s × 1 s (^1^D × ^2^D). The overlap was 50% in all directions, i.e., the tile borders were located in the center of the neighboring tiles. For each tile and each *m*/*z* value, a summed intensity was calculated. To retain the entire information, no data filtering was applied at this point, and the software parameters “area”, “height”, and “width” were thus set to zero.

The output was a set of 121,296 “features”. Each feature was defined by three parameters (^1^*t*_R_ of the tile center, ^2^*t*_R_ of the tile center, *m*/*z* value) and the corresponding intensities in the individual GC×GC–TOFMS runs. These data were imported into ChromCompare+, and for each GC×GC–TOFMS run data set, the truffle species was manually added. The *m*/*z* intensities in each GC×GC–TOFMS run data set were normalized using feature F41817, which originated from the internal standard (^2^H_8_)naphthalene as a reference (cf. Supporting Information, Table S2). The normalized *m*/*z* intensities were subjected to a log10 transformation followed by a principal component analysis (PCA). In the PCA, both biological replicates (2 or 3 workups) and technical replicates (3 GC×GC–TOFMS runs) were not averaged but treated independently. Using the feature discovery tool in ChromCompare+, the number of features included in the PCA was stepwise reduced.

### GC–MS Quantitation of Bis(methylsulfanyl)methane and Furan-2(5*H*)-one

Dichloromethane (20 mL), the internal standards (^2^H_8_)bis(methylsulfanyl)methane (0.0743–14.9 μg) and (^2^H_2_)furan-2(5*H*)-one (0.138–2.76 μg), and anhydrous sodium sulfate (1.5 g) were added to cryomilled truffles (0.5 g) under ice-cooling, and the mixture was homogenized with an Ultra-Turrax T25 (IKA) at 13500 rpm for 20 s. Extraction, filtration, aSAFE, and concentration were performed as detailed before. At a concentrate volume of ∼1 mL, 100 μL was sampled for the GC–MS analysis of the higher concentrated marker compound. The remaining ∼900 μL was further concentrated to a final volume of ∼100 μL by using a Bemelmans microdistillation device,^31^ and this portion was used for the analysis of the lower concentrated marker compound. For each truffle sample, a triplicate workup was performed.

The truffle volatile isolates were stored in amber glass vials at −24 °C prior to analysis with a GC–MS system consisting of a 7890B gas chromatograph, a Combi PAL autosampler, a multimode inlet used in splitless mode, a DB-FFAP column, 30 m × 0.25 mm i.d., 0.25 μm film thickness, and a Saturn 220 ion trap mass spectrometer (Agilent). Helium at a constant flow of 1.2 mL/min served as the carrier gas. The injection volume was 2 μL. For the analysis of bis(methylsulfanyl)methane, the initial inlet temperature was 40 °C (2 min), followed by a gradient of 6 °C/min to 118 °C, a gradient of 40 °C/min to 230 °C, and a final gradient of 900 °C/min to 250 °C. The initial oven temperature was 40 °C (2 min), followed by a gradient of 6 °C/min to 118 °C and a gradient of 40 °C/min to a final temperature of 230 °C (5 min). A rather mild temperature program in the injector that paralleled the temperature program in the oven was found to be crucial to avoid thermal degradation of the target compound during injection. The analysis of furan-2(5*H*)-one was performed with an initial inlet temperature of 40 °C, followed by a gradient of 900 °C/min to 250 °C (5 min), and a final gradient of 900 °C/min to 280 °C. The initial oven temperature was 40 °C (2 min), followed by a gradient of 6 °C/min to 166 °C and a gradient of 40 °C/min to a final temperature of 230 °C (5 min). Mass spectra were generated in EI mode at 70 eV with a scan range of *m*/*z* 35–250. Data analysis was performed using MS Workstation 7.0.2 software (Agilent).

Peak areas of the analyte and the respective internal standard were obtained from extracted-ion chromatograms (EICs) of the characteristic quantifier ions. The concentration of the marker compound was calculated from the acquired peak areas of the analyte and the internal standard, the amount of truffle sample used for the workup, and the amount of internal standard added by applying a calibration line equation. The calibration line equation was obtained by linear regression applied to the data obtained from the GC–MS analysis of analyte/standard mixtures in different concentration ratios. The quantifier ions and the calibration line equations are summarized in the Supporting Information file, Table S3. The individual concentrations obtained from the triplicate workups and the standard deviations are available in the Supporting Information file, Tables S4 and S5.

## Results and Discussion

### Screening for Marker Compounds

The untargeted volatilomics approach selected for the marker compound screening combined aSAFE for volatile isolation and comprehensive two-dimensional gas chromatography–mass spectrometry with a GC×GC–TOFMS instrument for volatile analysis. SAFE is a markedly gentle isolation technique that preserves the composition of the volatile fraction during isolation.^28,32^ aSAFE, the automated variant of SAFE, additionally provides high reproducibility due to the electronically controlled switching technique, which operates with fixed settings independently of the user.^28^ Finally, GC×GC–TOFMS combines an extraordinarily high separation efficiency on the chromatographic level with a high linear range on the mass spectrometric level, reported to allow for the semiquantitation of over 1000 compounds in a single run.^33^

The approach was applied to 17 *T. magnatum* and 6 *T. borchii* samples of confirmed authenticity. Each sample was subjected to duplicate or triplicate workup (depending on sample size), and each truffle volatile isolate was analyzed in triplicate by GC×GC–TOFMS analysis. The GC×GC–TOFMS raw data were subjected to retention time alignment and feature detection. The result was >100,000 features, which reflected the complexity of the volatile fraction of the truffle samples. Intensity data were normalized and subjected to log10 transformation followed by PCA with stepwise reduction of the number of features. Finally, differentiation of the two white truffle species, *T. magnatum* and *T. borchii*, was achieved based on only five features. For each of these, application of a Welch’s *t* test to the intensity values showed mean values that significantly differed between *T. magnatum* and *T. borchii* (*p*-values < 0.001; cf. Supporting Information, Table S1).

**Figure 1:**
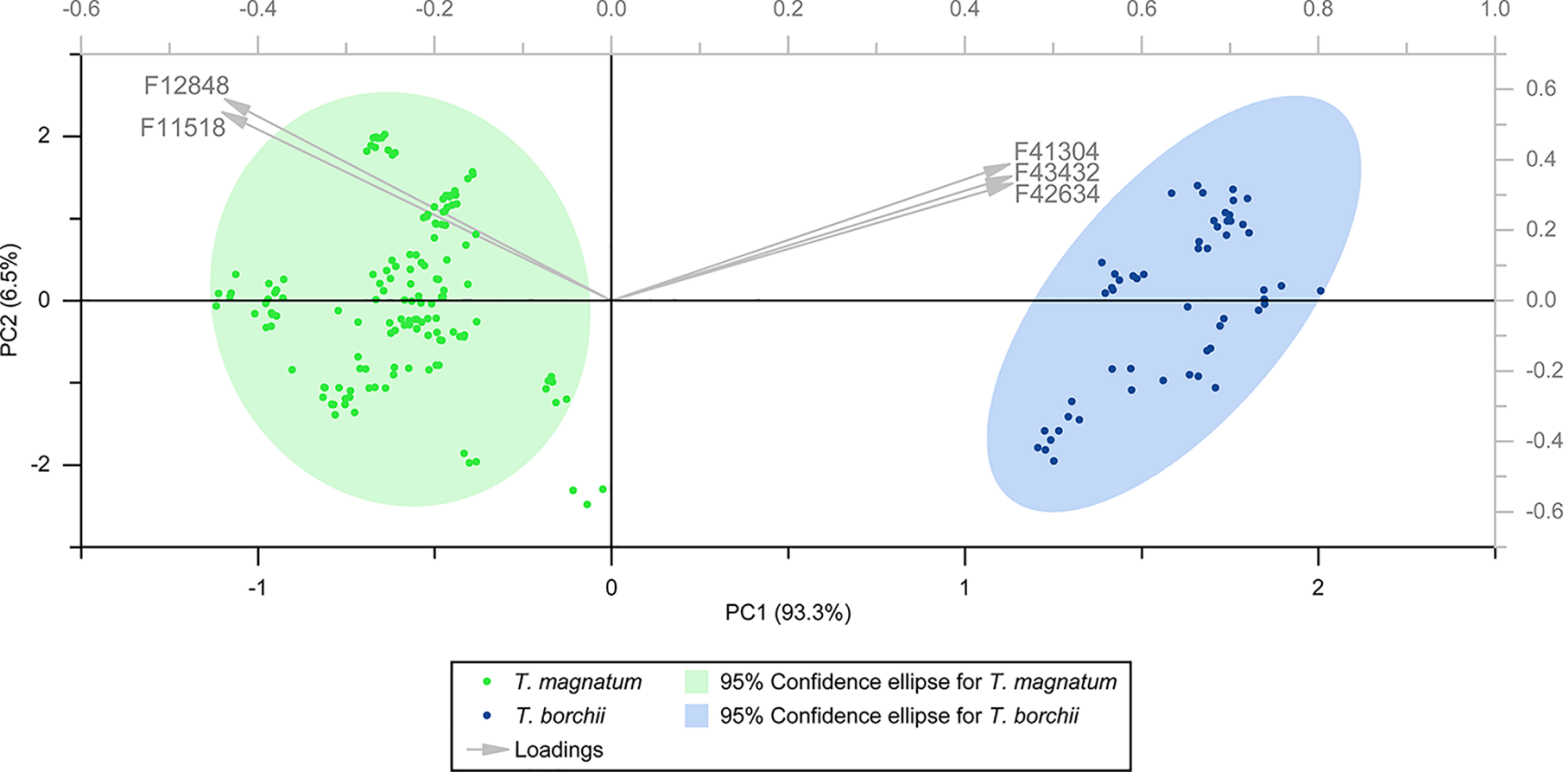
Biplot of the principal component analysis based on the five most relevant features obtained in the untargeted marker screening approach.

The biplot of the PCA based on the five features is depicted in Figure 1. The two species were clearly separated into two distinct clusters. The features characterizing the *T. magnatum* samples were F11518 and F12848, whereas the features characterizing the *T. borchii* samples were F41304, F42634, and F43432. The two clusters associated with the *T. magnatum* and *T. borchii* samples were clearly separated along principal component 1 (PC1). PC1 alone accounted for 93.3% of the total variance. In combination with PC2 (6.5%), 99.8% of the total variance was covered. Three *T. magnatum* data points were not included in the 95% confidence ellipse of the cluster. However, we did not consider them as outliers as all three were derived from the same biological sample and thus obviously reflected the biological variability within *T. magnatum*.

**Figure 2:**
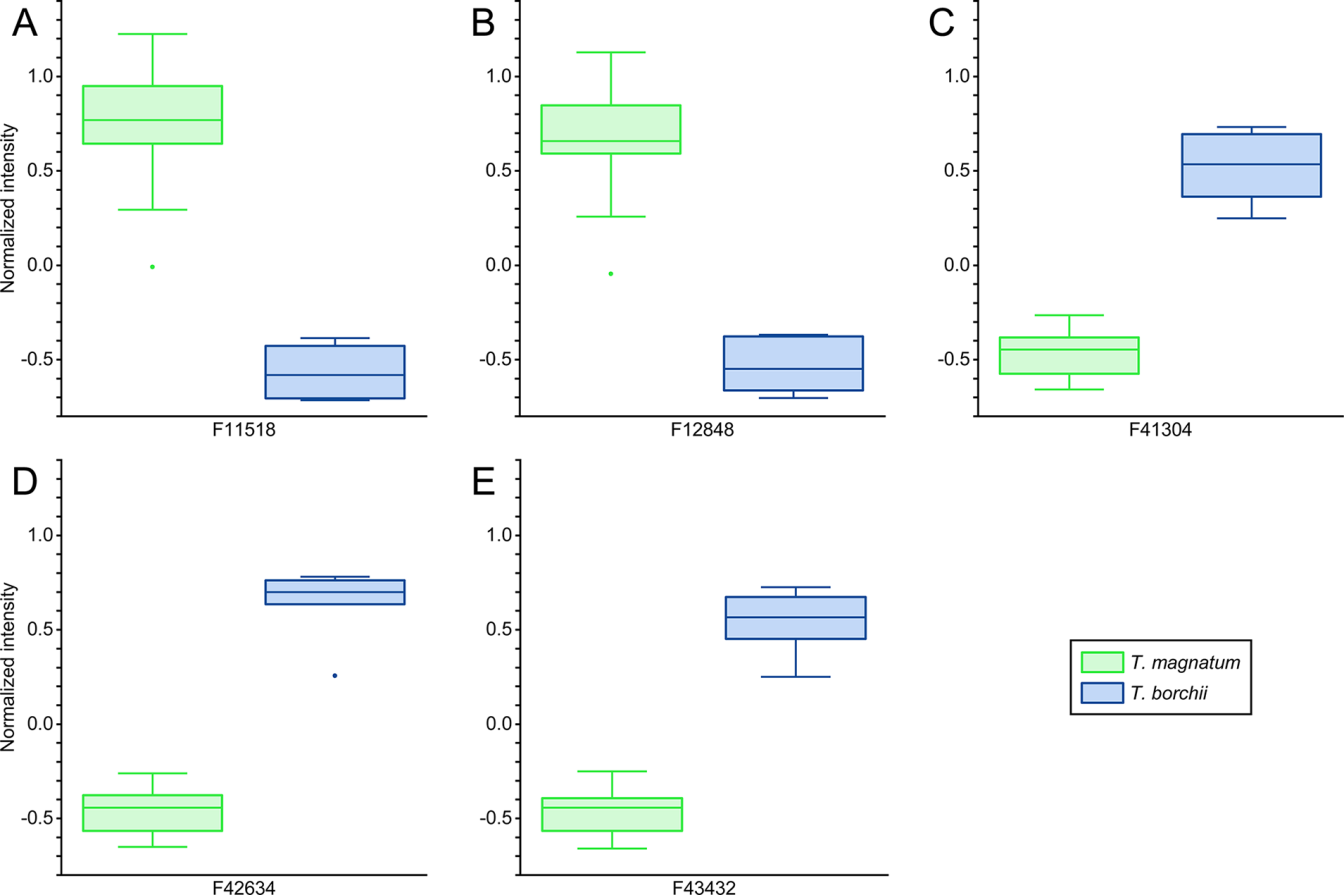
Box plots showing the semiquantitative intensity values of the five most relevant features (A–E) obtained in the untargeted marker screening approach.

An alternative visualization of the differentiation between *T. magnatum* and *T. borchii* on the basis of the five previously identified features is depicted in Figure 2. For each feature, the normalized intensities associated with *T. magnatum* and *T. borchii* were displayed as box plots. Features F11518 and F12848 showed higher intensities in *T. magnatum*, and features F41304, F43432, and F42634 showed higher intensities in *T. borchii*. Most importantly, in all five features, the *T. magnatum* and *T. borchii* data were well separated with no overlap.

As the next step, the compounds behind the five crucial features were identified. The feature characteristics (^1^*t*_R_, ^2^*t*_R_, and *m*/*z* values) are summarized in Table S2. Features F11518 and F12848, characterizing *T. magnatum*, showed the same *m*/*z* value (61) and were derived from neighboring tiles. This suggested that both features originated from a single compound. Likewise, features F41304, F43432, and F42634, characterizing *T. borchii*, all showed an *m*/*z* value of 55 and their tiles were also adjacent. Thus, there was, most probably, also only one underlying compound. The exact positions of the two crucial compounds in the GC×GC chromatograms were determined using EICs based on the features’ *m*/*z* values, 61 and 55, respectively, and the associated mass spectra were compared to database spectra.^34^ In both cases, the database search returned hits with match and reverse match factors >900 and probabilities >97%, suggesting that the compound characterizing *T. magnatum* was bis(methylsulfanyl)methane and the compound characterizing *T. borchii* was furan-2(5*H*)-one. GC×GC–TOFMS analysis of authentic reference compounds confirmed the structure assignments: when analyzed under the same conditions, the reference compounds returned the same values for ^1^*t*_R_, ^2^*t*_R_, and identical EI mass spectra as obtained from the truffle volatile isolates (Supporting Information, Figures S1 and S2). In summary, the untargeted screening suggested bis(methylsulfanyl)methane as a marker compound for *T. magnatum* and furan-2(5*H*)-one as a marker compound for *T. borchii*.

### Verification of the Marker Compounds by Exact Quantitation

Although the marker compound screening was successful, it must be considered that the differentiation between *T. magnatum* and *T. borchii* depicted in Figures 1 and 2 was based on semiquantitative data only. Furthermore, GC×GC–TOFMS analysis is expensive and unsuitable for routine analysis in truffle companies or commercial laboratories. Consequently, we aimed to verify the marker compound properties of bis(methylsulfanyl)methane and furan-2(5*H*)-one by a targeted approach that combines the accuracy of the results with a more straightforward GC–MS system. Thus, stable isotopologues of the target compounds were used as internal standards. This fully compensates for losses during workup and analysis and allows for the acquisition of accurate quantitative data independent of workup details and instrumental platforms. Furthermore, an economic one-dimensional system readily available in quality control laboratories was used for the GC–MS measurements.

**Figure 3:**
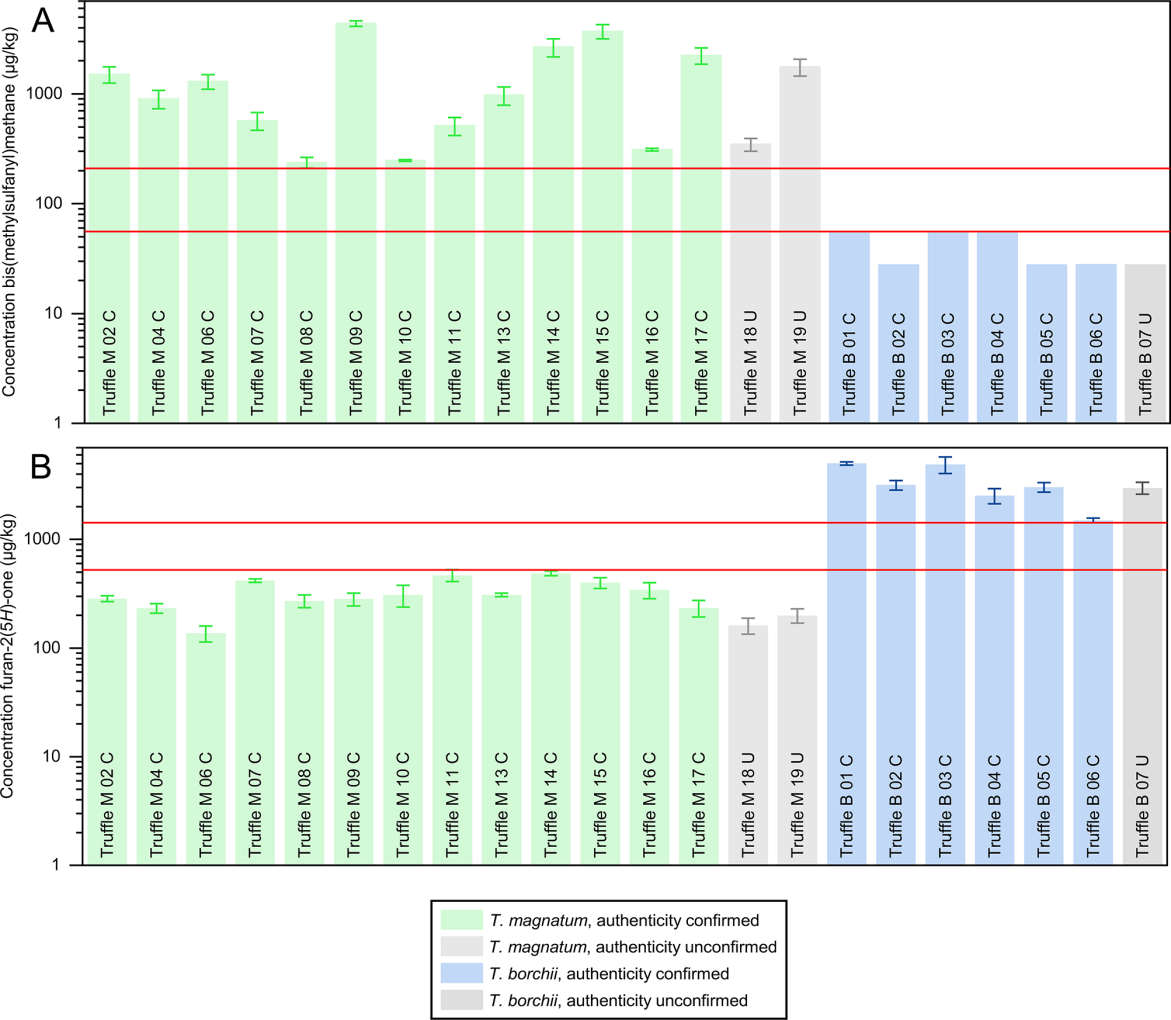
Concentrations of bis(methylsulfanyl)methane (A) and furan-2(5*H*)-one (B) in samples of *Tuber magnatum* and *Tuber borchii* with confirmed and unconfirmed authenticity.

The targeted quantitation approach was applied to 13 *T. magnatum* samples and six *T. borchii* samples of confirmed authenticity, all of which had previously been included in the untargeted marker compound screening. Three additional samples whose authenticity was not confirmed were analyzed, among which two were labeled as *T. magnatum* and one as *T. borchii*. Each sample was subjected to a triplicate workup. The results (Figure 3) confirmed the outcome of the semiquantitative analyses (cf. Figure 2). The concentration of bis(methylsulfanyl)methane (Figure 3A) in the 13 confirmed *T. magnatum* samples ranged from 237 ± 27 to 4360 ± 260 μg/kg (Supporting Information, Table S4). By contrast, bis(methylsulfanyl)methane was undetectable in the six confirmed *T. borchii* samples. Integration of the background noise in these samples indicated theoretical maximum bis(methylsulfanyl)methane concentrations below 56 μg/kg. Thus, the bis(methylsulfanyl)methane concentration in the *T. magnatum* samples was consistently higher than in the *T. borchii* samples, with an empty window of 154 μg/kg between the two data sets when the error bars were considered (Figure 3A, range between the red lines). This corresponded to a factor of 3.75. Likewise, the concentration of furan-2(5*H*)-one in the six confirmed *T. borchii* samples was consistently higher than that in the *T. magnatum* samples (Figure 3B). Whereas the values in the *T. borchii* samples ranged from 1490 ± 80 to 5010 ± 160 μg/kg, the values in the *T. magnatum* samples ranged only from 137 ± 23 to 487 ± 25 μg/kg (Supporting Information, Table S5). Considering the error bars, this corresponded to an empty window between the two concentration intervals of 898 μg/kg (Figure 3B, range between the red lines), resulting in a factor of 2.75.

**Figure 4:**
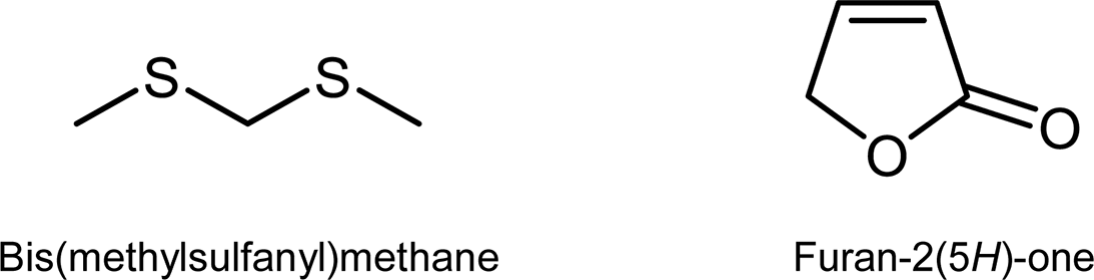
Marker compounds bis(methylsulfanyl)methane and furan-2(5*H*)-one characterizing the white truffle species *Tuber magnatum* and *Tuber borchii*, respectively.

In summary, the data showed that the quantitation of the two volatile marker compounds bis(methylsulfanyl)methane and furan-2(5*H*)-one (Figure 4) is a suitable analytical approach to distinguish between *T. magnatum* and *T. borchii*. This conclusion was supported by the results obtained from the samples without confirmed authenticity, which were purchased on the Internet. Both the two samples sold as *T. magnatum* and the sample sold as *T. borchii* showed concentrations of bis(methylsulfanyl)methane and furan-2(5*H*)-one in the expected ranges, indicating that they were correctly labeled (Figure 3, gray bars). In view of the low requirements regarding instrumentation, the method is directly available to be used in routine analysis for the objective species differentiation of *T. magnatum* and *T. borchii*.

## Supporting Information Available

*t*- and *p*-Values associated with the difference in the intensity values of the five crucial features between *T. magnatum* and *T. borchii*; characteristics of the five crucial features and the internal standard as obtained from the GC×GC–TOFMS screening; stable isotopically substituted internal standards, quantifier ions, and calibration lines used in the targeted quantitation of the marker compounds; individual concentration values used for calculating the mean values and standard deviations; signals and mass spectra obtained for bis(methylsulfanyl)methane and furan-2(5*H*)-one in the truffle volatile isolates and from the respective reference compounds by GC×GC–TOFMS analysis (PDF)

## Abbreviations

^1^D: first dimension
^2^D: second dimension
^1^*t*_R_: retention time in the first dimension
^2^*t*_R_: retention time in the second dimension
aSAFE: automated solvent-assisted flavor evaporation
CIS: cooled injection system
EI: electron ionization
EIC: extracted-ion chromatogram
GC: gas chromatography
GC×GC–TOFMS: comprehensive two-dimensional gas chromatography–time-of-flight mass spectrometry
GC–MS: gas chromatography–mass spectrometry
HS–SPME: headspace solid-phase microextraction
LC: liquid chromatography
MS: mass spectrometry
*m*/*z*: mass-to-charge ratio
PC: principal component
PCA: principal component analysis
SAFE: solvent-assisted flavor evaporation
*t*_R_: retention time
