# Supplementary material for: Identification of Bis(methylsulfanyl)methane and Furan-2(5H)-one as Volatile Marker Compounds for the Differentiation of the White Truffle Species Tuber magnatum and Tuber borchii
Source: J Agric Food Chem. 2024 Apr 17;72(17):10023–30. doi: 10.1021/acs.jafc.4c00714 (PMC11066859; doi:10.1021/acs.jafc.4c00714)
Supplement: Supplementary file 1 — jf4c00714_si_001.pdf [file jf4c00714_si_001.pdf]

Supporting Information

Identification of bis(methylsulfanyl)methane and furan-2(5*H*)-one as volatile marker compounds for the differentiation of the white truffle species *Tuber magnatum* and *Tuber borchii*

Philipp Schlumpberger[1,2] and Martin Steinhaus[2,1,*]

[1] Technical University of Munich, TUM School of Natural Sciences, Department of Chemistry, Lichtenbergstraße 4, 85748 Garching, Germany

[2] Leibniz Institute for Food Systems Biology at the Technical University of Munich (Leibniz-LSB@TUM), Lise-Meitner-Straße 34, 85354 Freising, Germany

*E-mail: martin.steinhaus@tum.de

Overview

Table S1. ***t*-Values and Associated *p*-Values Calculated by Welch's *t*-Tests from the Intensity Values of the Five Crucial Features Obtained from the GC×GC–TOFMS Screening**

Table S2. **Characteristics of the Five Crucial Features and the Internal Standard as Obtained from the GC×GC–TOFMS Screening**

Table S3. **Stable Isotopically Substituted Internal Standards, Quantifier Ions, and Calibration Lines Used in the GC–MS Quantitation of Bis(methylsulfanyl)-methane and Furan-2(5*H*)-one**

Table S4. **Concentrations of Bis(methylsulfanyl)methane in White Truffle Samples of Confirmed and Unconfirmed Authenticity**

Table S5. **Concentrations of Furan-2(5*H*)-one in White Truffle Samples of Confirmed and Unconfirmed Authenticity**

Figure S1. **Relevant Chromatogram Section and Mass Spectrum Obtained for Bis(methylsulfanyl)methane by GC×GC–TOFMS Analysis: *T. magnatum* Volatile Isolate (A) vs. Reference Compound (B)**

Figure S2. **Relevant Chromatogram Section and Mass Spectrum Obtained for Furan-2(5*H*)-one by GC×GC–TOFMS Analysis: *T. borchii* Volatile Isolate (A) vs. Reference Compound (B)**

Table S1. *t*-Values and Associated *p*-Values Calculated by Welch's *t*-Tests from the Intensity Values of the Five Crucial Features Obtained from the GC×GC–TOFMS Screening

feature ID	*t*-value	degree of freedom	*p*-value
**F11518**	46.9	166	2.71×10^{-97}
**F12848**	44.8	150	2.99×10^{-88}
**F41304**	36.9	77.5	1.21×10^{-50}
**F42634**	38.5	73.4	4.22×10^{-50}
**F43432**	38.6	79.9	3.90×10^{-53}

Table S2. Characteristics of the Five Crucial Features and the Internal Standard as Obtained from the GC×GC–TOFMS Screening

feature ID	${}^{1}t_R$ (min)	${}^{2}t_R$ (s)	*m*/*z*	underlying compound
**F11518**	10	2.5	61	bis(methylsulfanyl)methane
**F12848**	10	2.0	61	bis(methylsulfanyl)methane
**F41304**	24	2.5	55	furan-2(5*H*)-one
**F41817**	24	3.5	136	(${}^{2}H_8$)naphthalene (internal standard)
**F42634**	24	2.0	55	furan-2(5*H*)-one
**F43432**	25	2.5	55	furan-2(5*H*)-one

Table S3. Stable Isotopically Substituted Internal Standards, Quantifier Ions, and Calibration Lines Used in the GC–MS Quantitation of Bis(methylsulfanyl)methane (1) and Furan-2(5*H*)-one (2)

	internal standard	quantifier ions (*m*/*z*)		calibration line equation	R^2
		analyte	standard		
1	(${}^{2}H_8$)bis(methylsulfanyl)methane	61	66	$y = 1.101x + 0.005$[a]	1.000
2	(${}^{2}H_2$)furan-2(5*H*)-one	84	86	$y = 1.276x + 0.100$[b]	0.999

[a]y = peak area counts standard / peak area counts analyte; x = concentration standard (µg/kg) / concentration analyte (µg/kg). [b]y = peak area counts analyte / peak area counts standard; x = concentration analyte (µg/kg) / concentration standard (µg/kg).

Table S4. Concentrations of Bis(methylsulfanyl)methane in White Truffle Samples of Confirmed and Unconfirmed Authenticity

sample[a]	concentration (µg/kg)			
	experiment 1	experiment 2	experiment 3	mean ± SD (CV)[b]
truffle B 01 C	≤51.0[c]	≤55.4[c]	≤51.4[c]	
truffle B 02 C	≤27.5[c]	≤27.6[c]	≤27.5[c]	
truffle B 03 C	≤55.6[c]	≤55.4[c]	≤55.0[c]	
truffle B 04 C	≤55.2[c]	≤55.6[c]	≤55.3[c]	
truffle B 05 C	≤27.4[c]	≤27.7[c]	≤27.6[c]	
truffle B 06 C	≤27.6[c]	≤27.8[c]	≤27.5[c]	
truffle B 07 U	≤27.6[c]	≤27.6[c]	≤27.7[c]	
truffle M 02 C	1230	1560	1730	1510 ± 250 (17%)
truffle M 04 C	708	1030	972	903 ± 172 (19%)
truffle M 06 C	1100	1490	1300	1300 ± 195 (15%)
truffle M 07 C	694	512	509	572 ± 106 (19%)
truffle M 08 C	235	211	265	237 ± 27 (11%)
truffle M 09 C	4620	4360	4110	4360 ± 260 (6%)
truffle M 10 C	251	248	243	247 ± 4 (2%)
truffle M 11 C	543	594	406	514 ± 97 (19%)
truffle M 13 C	943	808	1170	974 ± 183 (19%)
truffle M 14 C	3180	2630	2180	2660 ± 500 (19%)
truffle M 15 C	3100	4180	3860	3710 ± 560 (15%)
truffle M 16 C	305	317	178[d]	311 ± 8 (3%)
truffle M 17 C	2670	2060	1980	2240 ± 380 (17%)
truffle M 18 U	294	366	380	347 ± 46 (13%)
truffle M 19 U	1660	1520	2100	1760 ± 300 (17%)

[a]B, *T. borchii*; M, *T. magnatum*; C, authenticity confirmed; U, authenticity unconfirmed. [b]SD, standard deviation; CV, coefficient of variation. [c]No analyte peak was observed; values were derived from the integration of the background noise and the highest value among the 3 experiments was finally assigned to the truffle sample [d]Value was considered as outlier based on the Nalimov test and thus excluded from further calculations.

Table S5. Concentrations of Furan-2(5*H*)-one in White Truffle Samples of Confirmed and Unconfirmed Authenticity

sample[a]	concentration (µg/kg)			
	experiment 1	experiment 2	experiment 3	mean ± SD (CV)[b]
truffle B 01 C	4880	5190	4950	5010 ± 160 (3%)
truffle B 02 C	3410	2800	3300	3170 ± 330 (10%)
truffle B 03 C	5770	4090	4810	4890 ± 840 (17%)
truffle B 04 C	2440	2180	2960	2530 ± 400 (16%)
truffle B 05 C	3330	3050	2710	3030 ± 310 (10%)
truffle B 06 C	1510	1560	1410	1490 ± 80 (5%)
truffle B 07 U	2950	2620	3360	2980 ± 370 (12%)
truffle M 02 C	302	285	267	285 ± 18 (6%)
truffle M 04 C	215	224	260	233 ± 24 (10%)
truffle M 06 C	163	122	125	137 ± 23 (17%)
truffle M 07 C	408	429	189[c]	419 ± 15 (4%)
truffle M 08 C	230	287	298	272 ± 37 (13%)
truffle M 09 C	309	255	1340[c]	282 ± 38 (14%)
truffle M 10 C	232	370	323	308 ± 70 (23%)
truffle M 11 C	404	478	520	467 ± 59 (13%)
truffle M 13 C	300	322	304	309 ± 12 (4%)
truffle M 14 C	476	470	516	487 ± 25 (5%)
truffle M 15 C	384	363	450	399 ± 45 (11%)
truffle M 16 C	278	388	360	342 ± 57 (17%)
truffle M 17 C	188	246	266	233 ± 41 (17%)
truffle M 18 U	177	176	130	161 ± 27 (17%)
truffle M 19 U	232	174	192	199 ± 30 (15%)

[a]B, *T. borchii*; M, *T. magnatum*; C, authenticity confirmed; U, authenticity unconfirmed. [b]SD, standard deviation; CV, coefficient of variation. [c]Value was considered as outlier based on the Nalimov test and thus excluded from further calculations.

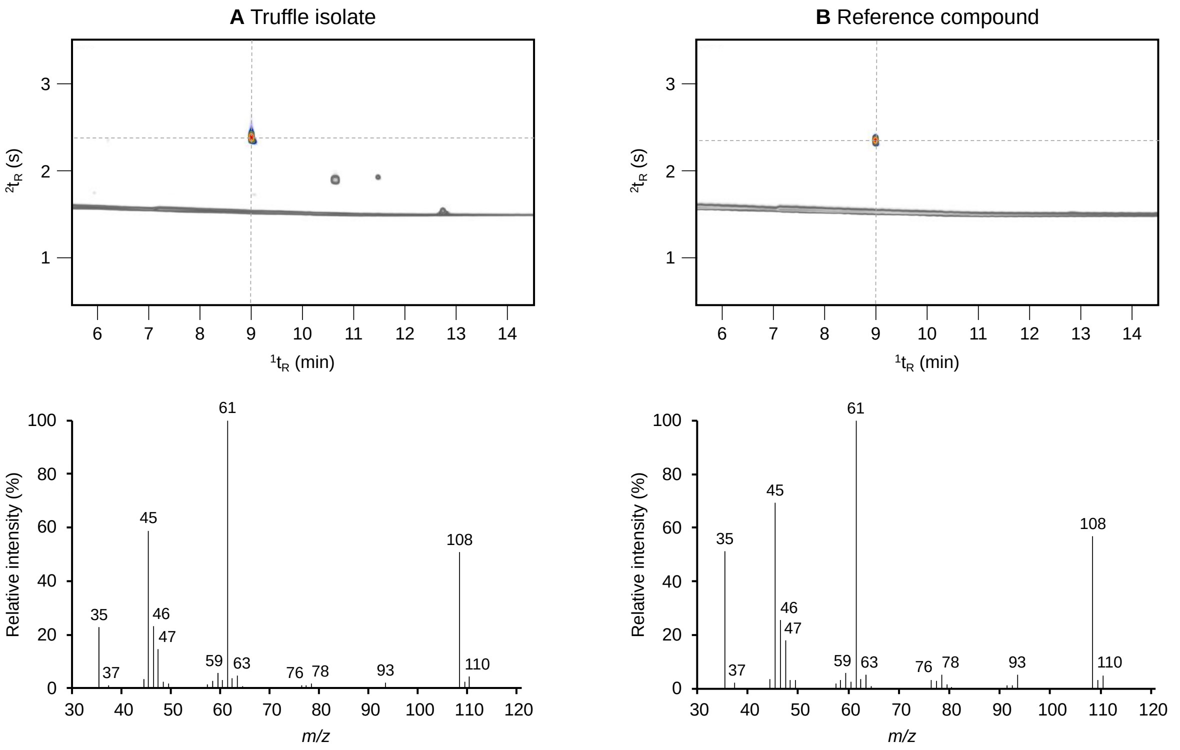

Figure S1. Relevant Chromatogram Section and Mass Spectrum Obtained for Bis(methylsulfanyl)methane by GC×GC–TOFMS Analysis: *T. magnatum* Volatile Isolate (A) vs. Reference Compound (B)

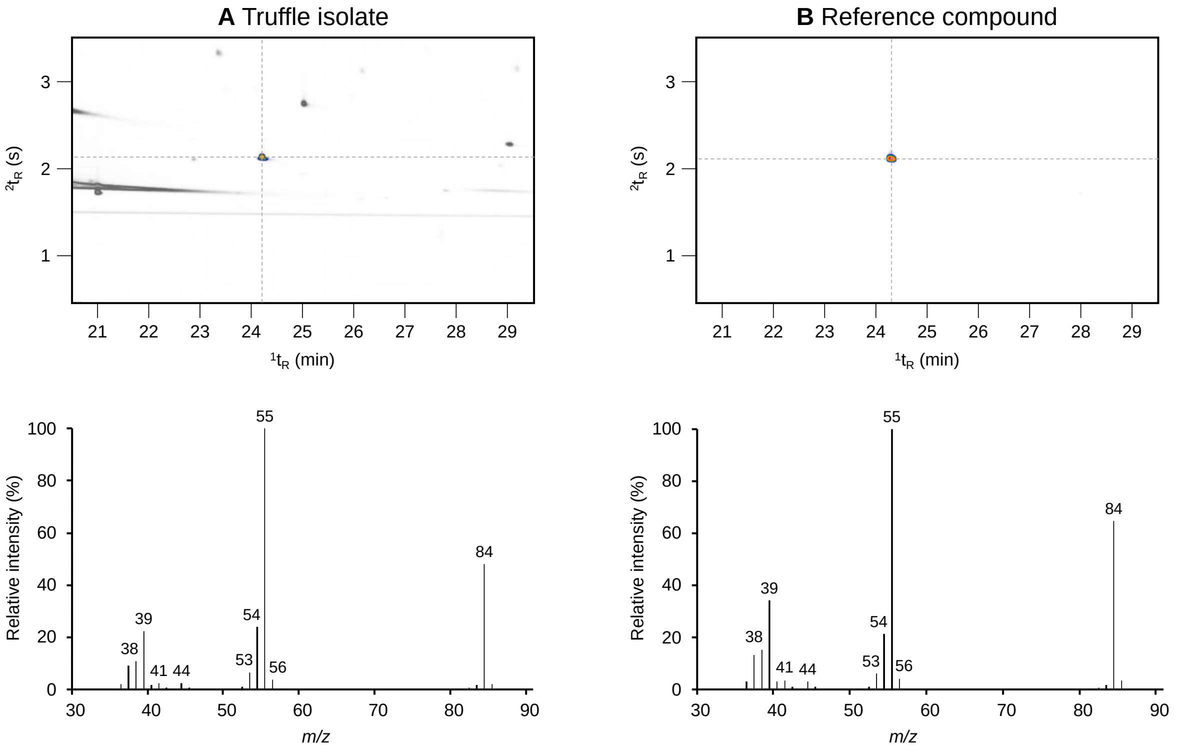

Figure S2. Relevant Chromatogram Section and Mass Spectrum Obtained for Furan-2(5*H*)-one by GC×GC–TOFMS Analysis: *T. borchii* Volatile Isolate (A) vs. Reference Compound (B)
